# Improving Radiology Report Error Detection Using a Multipass Large Language Model: Framework Development and Validation

**DOI:** 10.2196/87368

**Published:** 2026-06-04

**Authors:** Songsoo Kim, Seungtae Lee, See Young Lee, Joonho Kim, Keechan Kan, Hyunji Lee, Dukyong Yoon

**Affiliations:** 1 Department of Radiology, Seoul National University Hospital, Seoul, Republic of Korea; 2 Department of Radiology, Gangnam Severance Hospital, Seoul, Republic of Korea; 3 Department of Internal Medicine, Gangnam Severance Hospital, Seoul, Republic of Korea; 4 Department of Neurology, Severance Hospital, Seoul, Republic of Korea; 5 Department of Surgery, Samsung Medical Center, Seoul, Republic of Korea; 6 Department of Obstetrics and Gynecology, Kangbuk Samsung Hospital, Seoul, Republic of Korea; 7 Department of Biomedical Systems Informatics, College of Medicine, Yonsei University, 101-604, Seoul, 03687, Republic of Korea, 82 31-5189-8450, 82 31-5189-8450; 8 Institute for Innovation in Digital Healthcare, Severance Hospital, Seoul, Republic of Korea

**Keywords:** large language models, radiology report, quality assurance, error detection, human-in-the-loop

## Abstract

**Background:**

Large language model (LLM) proofreaders for radiology reports generate many false positives (FPs) due to the low prevalence of errors.

**Objective:**

This study aimed to determine whether an optimized LLM framework could improve both precision and cost-efficiency without compromising error detection capability.

**Methods:**

In this retrospective study, 1000 radiology reports (radiography, ultrasonography, computed tomography, and magnetic resonance imaging; 250 each) were sampled from the Medical Information Mart for Intensive Care III database. Two public chest radiography corpora (CheXpert and Open-i) served as external test sets. Three LLM frameworks were evaluated: single-prompt detector (framework 1); report extractor plus single-prompt detector (framework 2); and extractor, detector, and FP verifier (framework 3). Precision for each framework was assessed using positive predictive value (PPV) and detected errors per 1000 reports. Overall efficiency was estimated using model inference costs and reviewer labor costs.

**Results:**

PPV increased from 0.063 (95% CI 0.036‐0.101) in framework 1 to 0.079 (95% CI 0.049‐0.118) in framework 2 and 0.159 (95% CI 0.090‐0.252) in framework 3 (*P*<.001). Despite improved PPV, detected errors remained stable (detected errors per 1000 reports: 12‐14). Human review burden decreased from 192 to 88 reports. Framework 3 also reduced model inference costs to US $5.57 per 1000 reports (vs US $9.72 and US $6.85 for frameworks 1 and 2; 42.6% and 18.5% reductions, respectively). External validation confirmed similar improvements. Qualitative analysis revealed that remaining FPs in framework 3 were largely confined to cases requiring deep clinical context (clinically equivalent rephrasing: 53%; unsupported discrepancy assertions: 43%). By eliminating structural FPs (eg, section mismatches and lexical errors: 0%), the framework effectively shifted the quality assurance burden to a smaller set of ambiguous cases, enabling a targeted human-in-the-loop workflow.

**Conclusions:**

The multipass LLM improved the precision and cost-efficiency of radiology report error detection in real-world, low-error prevalence settings. The framework demonstrates the feasibility of synergistic artificial intelligence–radiologist collaboration and provides a cost-effective and scalable approach to artificial intelligence–assisted quality assurance in both radiological practice and research.

## Introduction

Large language models (LLMs) are increasingly being explored as an additional set of eyes for proofreading radiology reports [1,2]. However, when applied to real-world data, this extra “eye” often results in frequent false alarms. The precision of these models—also referred to as positive predictive value (PPV)—remains low because, despite “good” model specificity, the underlying error rate in clinical practice is extremely low. For example, in a setting with a 1% error prevalence, even a highly sensitive model with 90% specificity would still generate approximately 10 false alarms for every true error detected. In one experiment involving 10,000 real reports, GPT-4 achieved a PPV of only 6% despite good specificity, producing roughly 15 false alerts for each true error [3]. These excessive notifications contribute to alert fatigue among radiologists, prompting them to ignore subsequent warnings, hindering effective human-artificial intelligence (AI) collaboration, and—ironically—increasing the real-world workload [4].

Although continued advances in LLMs are expected to address these shortcomings, the anticipated gains present a double-edged sword in terms of overall utility [5]. Parameter scaling, task-specific fine-tuning, and deployment of multiagent systems [6,7] can certainly enhance model performance and clinical efficiency. However, these improvements come at substantial computational costs. Deploying multiagent systems, for example, routinely produces execution traces averaging more than 15,000 lines per session [8], while scaling to larger models dramatically increases resource demands—a recent study showed that LLaMA-3-70B incurred over 400 times the inference time and cost of a lightweight 3B-parameter model for radiology report structuring [9]. Consequently, AI-driven radiology report error detection faces a dual imperative: it must increase precision to reduce human workload while also remaining computationally feasible and cost-effective for routine clinical deployment.

Despite these limitations, previous studies still benchmark LLMs on error-inflated datasets and rarely explore strategies for improving PPV in low-error, real-world settings [1,10,11]. Similarly, strategies to improve operational cost-efficiency remain largely unexplored. Consequently, achieving clinical viability requires a framework capable of explicitly resolving the inherent trade-off between sensitivity and specificity observed in single-pass models [3].

To address these gaps, we present a multipass LLM framework designed to optimize both precision and efficiency. The pipeline (1) employs a lightweight report extractor to isolate clinical findings from structural noise, (2) applies stepwise reasoning to decouple error detection from verification, thereby mitigating the sensitivity-specificity trade-off, and (3) provides a user interface to facilitate rapid review of the model’s structured output by radiologists. A benchmark with 2 nonoptimized baselines was performed to quantify improvements in precision and efficiency.

## Methods

### Ethical Considerations

This study used only publicly available, deidentified radiology datasets (Medical Information Mart for Intensive Care III [MIMIC-III], CheXpert, and Open-i). Institutional Review Board approval and written informed consent were not required.

### Dataset Curation

Radiology reports were retrieved from the MIMIC-III database [12]. Using the “ISERROR” column in the database, which identifies physician-flagged erroneous notes, the study included only those reports that had been confirmed as error-free. To validate robustness across the heterogeneous nature of radiology reports and facilitate performance comparison across modalities, modality-level stratified random sampling was performed to construct a balanced primary test set comprising 1000 reports, with 250 reports each from radiography, ultrasonography, computed tomography (CT), and magnetic resonance imaging (MRI). An additional hold-out set of 50 predominantly radiography reports was reserved for prompt tuning and reviewer calibration. To assess the external generalizability of the proposed pipeline, 2 publicly available radiology report datasets—CheXpert and Open-i chest X-ray [13,14]—were used as external test sets. The characteristics of the final reports across all datasets are summarized in Table 1.

**Table 1.** Characteristics of MIMIC-III^a^, CheXpert, and Open-i radiology reports used in this study.

Characteristics	MIMIC-III				CheXpert (n=300)	Open-i (n=300)	*P* value^b^
	X-ray (n=250)	Ultrasound (n=250)	CT^c^ (n=250)	MRI^d^ (n=250)			
Characters, mean (SD)	1206.9 (367.6)	1419.6 (535.3)	2721.7 (1418.7)	2467.4 (1170.4)	525.9 (243.8)	334.7 (149.3)	<.001
Word count, mean (SD)	153.7 (53.2)	187.8 (78.2)	374.8 (208.7)	340.1 (170.5)	77.7 (37.1)	46.1 (22.6)	<.001
Sentence count, mean (SD)	28.4 (8)	32 (11.7)	59.6 (29.6)	52 (23.2)	13.9 (5.8)	10 (2.4)	<.001
History section, n (%)	250 (100)	248 (99.2)	250 (100)	250 (100)	240 (80)	300 (100)	<.001
Technique section, n (%)	24 (9.6)	32 (12.8)	216 (86.4)	201 (80.4)	14 (4.7)	0 (0)	<.001
Comparison section, n (%)	76 (30.4)	114 (45.6)	135 (54)	89 (35.6)	284 (94.7)	0 (0)	<.001

a MIMIC-III: Medical Information Mart for Intensive Care III.

b *P* values are from Kruskal-Wallis test (continuous variables) and Fisher exact test (categorical variables).

c CT: computed tomography.

d MRI: magnetic resonance imaging.

### Error Definition

The process of generating radiology reports can be divided into the following two main steps: (1) detecting abnormalities from images and (2) documenting the detected abnormalities [3]. Efforts have been made to use natural language processing models, including LLMs, to correct errors occurring in the second step [3,15-17]. Examples of these second-step errors may result from the misinterpretation of findings or the inclusion of factually inconsistent content in the report text. Following the classification proposed by Kim et al [3], errors were categorized into interpretive errors (addition, omission, and substitution) and factual errors (discrepancy in location/numerical measurement). The detailed description of error types is described in Table S1 in Multimedia Appendix 1.

### Proposed Framework and Experimental Design

**Figure 1.**
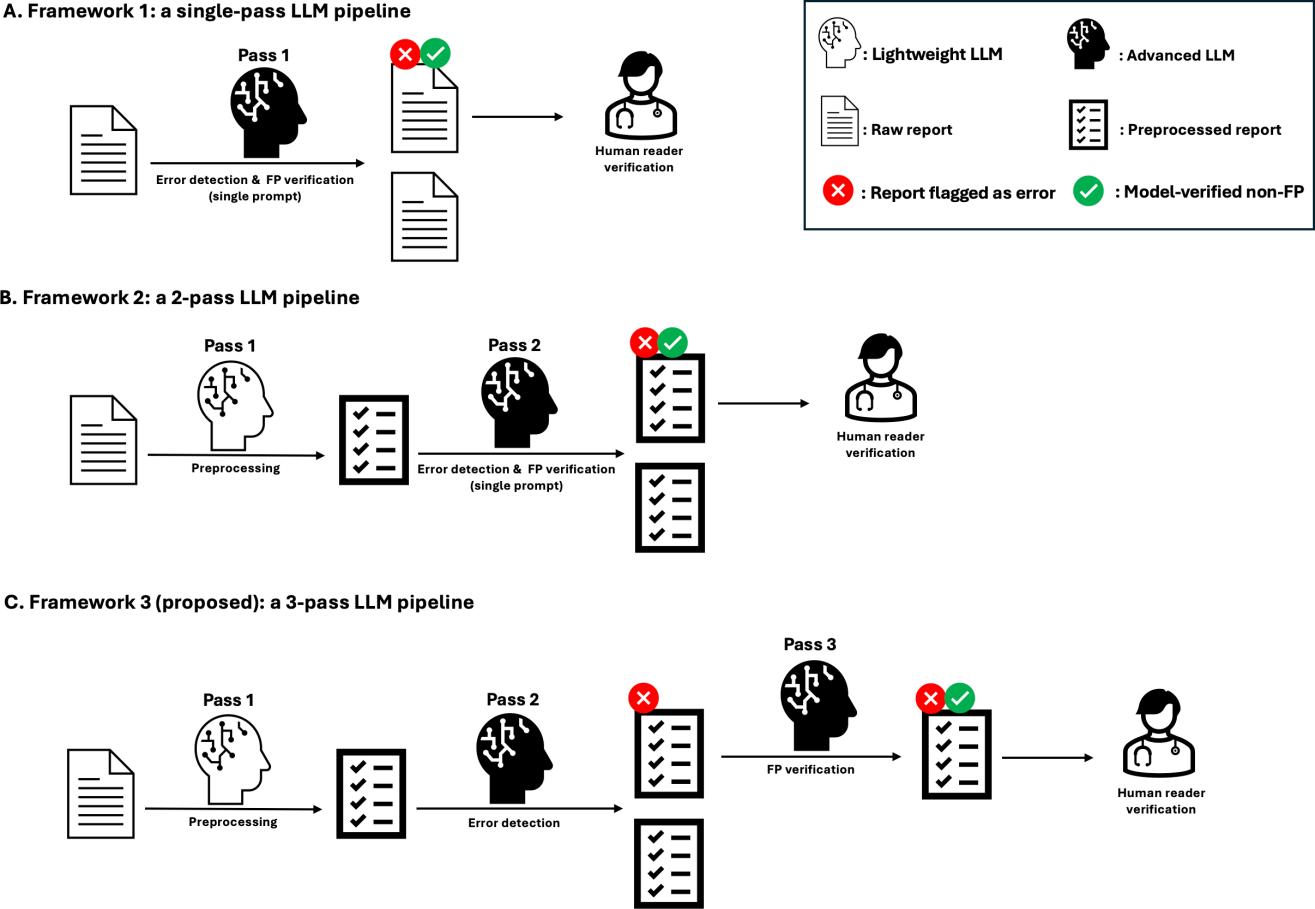
Experimental design of large language model (LLM) pipelines for radiology report error detection. In the single-pass framework (A), each report is processed once by an advanced LLM that simultaneously performs error detection and false-positive (FP) verification before reader’s review. In the 2-pass framework (B), a lightweight LLM first performs preprocessing, and an advanced LLM subsequently conducts combined detection and verification before reader’s review. In the proposed 3-pass framework (C), preprocessing is followed by error detection in a second pass and isolated FP verification in a third pass by an advanced LLM, prior to reader’s review.

Three LLM pipelines were compared (Figure 1). In framework 1, the original report was input directly into an advanced LLM, which performed both error detection and false-positive (FP) verification within a single prompt. In framework 2, a lightweight LLM first extracted and structured the relevant portion of the radiology report by removing content outside the “Findings” and “Impression” sections—such as clinical information, technique notes, and headers—and seamlessly merging any addenda into this section. The resulting structured Findings or Impression block was then passed to an advanced LLM, which performed combined error detection and FP verification in a single prompt. Framework 3 retained the preliminary extraction step but divided the downstream reasoning across 2 successive prompts: candidate errors were first enumerated and then reexamined to verify potential FPs.

**Figure 2.**
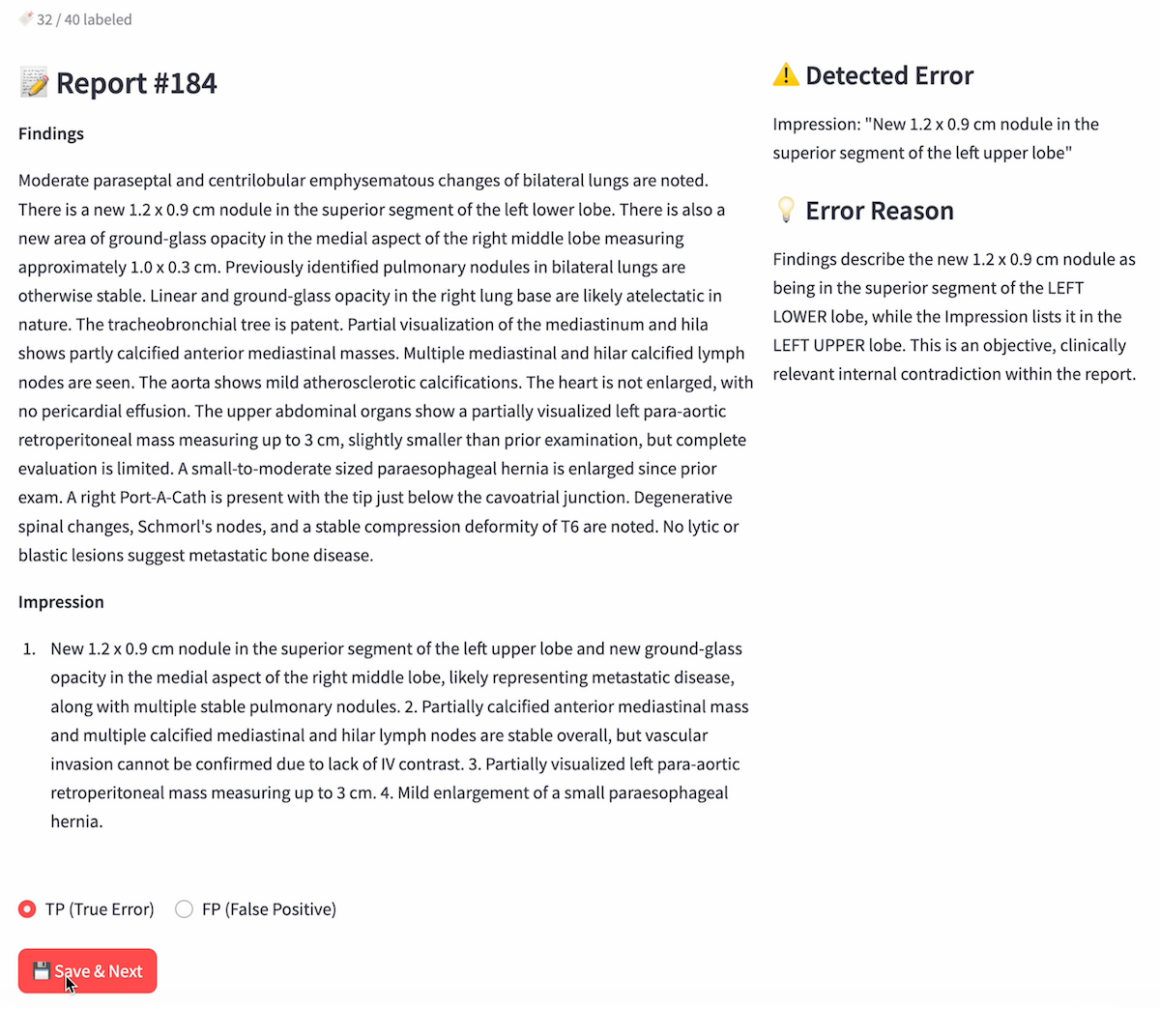
User interface for the multipass large language model (LLM) radiology report error detector. The review screen loads the preprocessed structured output, displaying the “Findings” and “Impression” sections in the left panel, while the right panel shows the detected error and the model-provided rationale using the error and error_reason keys. This structured layout enables reviewers to classify each finding as either a true positive (TP) or a false positive (FP) with a single click.

Final model responses were structured to include the radiology report, identified errors, and corresponding error reasoning [18]. The resulting outputs were streamed to a web-based quality assurance interface, which displayed the flagged report alongside the model’s error reasoning, allowing human reviewers to accept or reject each suggestion with a single click (Figure 2).

The lightweight LLM used in this experiment was executed by OpenAI’s GPT-4.1-nano, selected for its favorable cost-effectiveness, and the advanced LLMs were O3, chosen for their superior reasoning performance at the time of the study [19]. The 2 models have a 100-fold difference in cost per token. All pipelines were executed on the institution’s private Azure OpenAI Service, with each LLM API (application programming interface) call launched within an isolated API session. Detailed descriptions of the prompts and parameters are provided in Multimedia Appendix 1.

### Precision Evaluation

Each flagged report underwent a 2-step review. Two board-certified physicians (with 9 and 10 years of clinical experience, respectively) screened each model-generated alert against the original report using a standardized rubric aligned with our error taxonomy, labeling them as true positive (TP) or FP. Subsequently, 2 board-certified radiologists (with 8 and 9 years of clinical experience, respectively) adjudicated these labels to establish the final ground truth. Reviewer calibration on the held-out prompt-tuning set (n=50) showed an overall percent agreement of 94% (47/50). The performance of the framework was evaluated using PPV (PPV=TP/[TP+FP]) and the detected errors per 1000 reports (DE/1k=(TP/N)×1000), where N denotes the size of the test set. Here, TP refers to a model-flagged report in which a genuine error was confirmed, while FP refers to a flagged report that did not contain a true error. To analyze failure modes, 30 adjudicated FP alerts per framework were randomly sampled from the MIMIC-III test set (n=90). Each case was independently reviewed and classified into a 6-category taxonomy by 2 board-certified radiologists to track the evolution of error patterns.

### Operational Cost-Efficiency Evaluation

A cost-minimization analysis was conducted under the assumption of equal true error detection across all 3 frameworks. The estimated running cost was defined as the sum of (1) model inference costs and (2) reviewer labor costs [20]. Because the exact computational cost of the closed-source LLM could not be measured directly, we used per-token API charges as a proxy measure. This choice is supported by the evidence that electricity and graphics processing unit rental costs dominate token pricing in commercial LLMs [21,22]. Consequently, the model inference costs were calculated based on text volume and provider pricing rates (Eq  S1 in Multimedia Appendix 1).

Reviewer labor cost was approximated by multiplying the total number of reports sent for manual inspection—comprising both TPs and FPs—by the mean compensation paid per report (Eq S2 in Multimedia Appendix 1).

Reviewer labor costs were modeled using the median annual compensation for diagnostic radiologists (US $568,327) reported in the 2024 Medical Group Management Association (MGMA) compensation survey [23]. Assuming a standard 2,000-hour work year, this corresponds to an hourly rate of approximately US $284 or US $4.74 per minute. Consistent with review durations reported in prior literature [1,3], the analysis was performed by varying the review time per flagged report (30, 60, and 120 s) to estimate labor costs across different clinical scenarios.

The estimated running cost for each framework was therefore defined as the sum of the 2 components (Eq S3 in Multimedia Appendix 1) and is reported separately to permit direct operational cost-effectiveness comparisons. Formal derivations and the full set of symbols are provided in Multimedia Appendix 1.

### Statistical Analysis

Continuous variables are reported as mean (SD) when normally distributed (Shapiro-Wilk test, *P*>.05) and as median (IQR) otherwise. Categorical variables are summarized as counts and percentages. Between-dataset differences were assessed using the Kruskal–Wallis test for continuous variables and the Fisher exact test for categorical variables. PPV and DE/1k are expressed with 2-sided 95% exact (Clopper-Pearson) CIs [24].

For PPV comparisons, pairwise differences among the 3 frameworks were assessed using report-level paired-cluster bootstrap (10,000 replicates). Two-sided *P* values were extracted from the bootstrap distributions, and the family-wise error rate across the 3 comparisons was controlled using the Holm-Bonferroni procedure [25]. Modality-specific PPV analyses were regarded as exploratory and reported without multiplicity adjustment. When the frameworks followed a prespecified ordinal sequence, a Cochran-Armitage trend test was additionally applied to detect monotonic trends in PPV across the ordered groups.

For DE/1k comparisons, within-case differences among the 3 frameworks were evaluated using the exact McNemar test, with the family-wise error rate controlled via the Holm-Bonferroni procedure. When comparing 3 or more frameworks, an overall Cochran Q test was conducted; if significant, pairwise McNemar tests with Holm correction were performed. All tests were 2-tailed, with an α of .05.

The sample size was calculated based on the MIMIC-III dataset. The baseline PPV of the reference pipeline was assumed to be 6%, as previously reported [3]. A 2-fold improvement with the proposed pipeline was deemed a meaningful difference. Treating the comparison as a 2-sided test of the difference between 2 independent proportions and adopting *α*=.05 with a statistical power of 80%, a minimum of 716 reports was required. Consequently, the final sample of 1000 reports satisfied and exceeded this requirement, thereby ensuring adequate power for the primary hypothesis test. Analyses were performed in Python 3.11 using pandas 2.2.2, SciPy 1.12.0, and statsmodels 0.15.0 for statistical procedures and matplotlib 3.9.0 for visualization.

## Results

### Detected Errors and FP Cases

**Figure 3.**
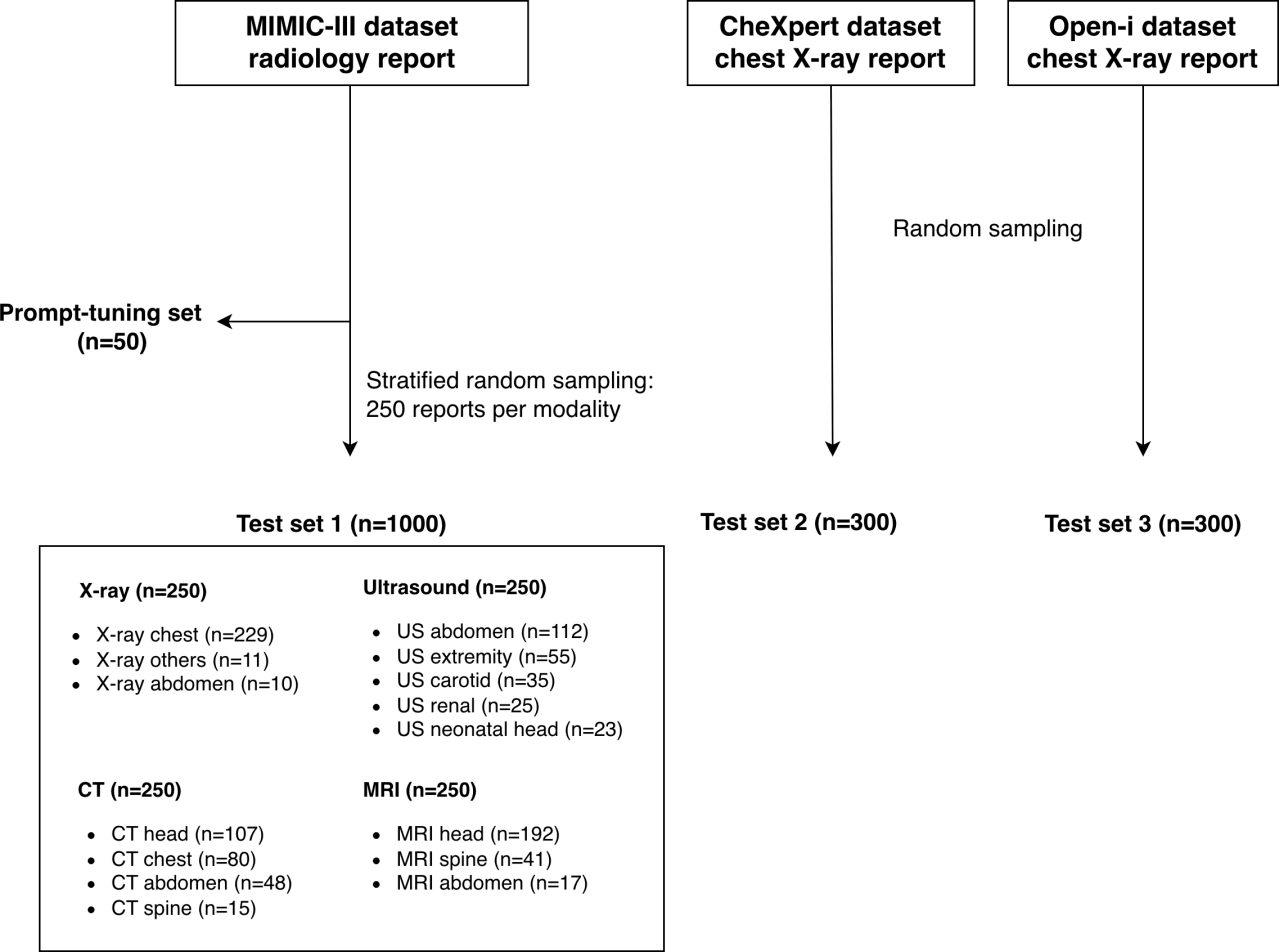
Flowchart of radiology report sampling from the Medical Information Mart for Intensive Care III (MIMIC-III), CheXpert, and Open-i datasets for prompt tuning and test set construction. CT: computed tomography; MRI: magnetic resonance imaging; US: ultrasound.

The true errors identified in each dataset are summarized in Table S4 in Multimedia Appendix 1. Fourteen errors were detected in the MIMIC-III dataset—2 in chest radiographs, 3 in carotid ultrasonography studies, 1 in a neonatal brain ultrasonography study, 3 in head CT scans, 2 in chest CT scans, and 3 in head MRI examinations (Figure 3). Two errors were found in both the CheXpert and Open-i datasets. The error distribution included discrepancies in anatomical location (9/18, 50%), omission (3/18, 17%), addition (3/18, 17%), and discrepancies in numeric measure (3/18, 17%); notably, errors detected by each framework followed a strict subset relationship, and the per-framework breakdown is provided in Table S5 in Multimedia Appendix 1.

Table 2 summarizes representative FP cases. FPs that occurred only in framework 1 arose chiefly from a rigid comparison of superficial header elements—such as date strings or minor omissions in the clinical history—with the body text, causing spurious contradiction flags. Once the header metadata had been removed, such false flags were not observed in framework 2 or 3. Framework 2 still produced many FPs because it compared sentences at the strict word level, with little regard for anatomical or contextual nuance. Framework 3 subsequently reexamined the candidate contradictions identified by framework 2 in the context of the full report and reclassified statements deemed acceptable in routine practice, thereby reducing the overall FP burden.

**Table 2.** Representative cases from the analysis of false positives across 3 different frameworks^a^.

Report excerpt	Framework 1	Framework 2	Framework 3	False-positive rationale
Header: “Comparison: 10/20.” “1. Compared to prior study from October 5th, 20, interval increase in...”	Error	No error	No error	Two legitimate comparison dates were interpreted as contradictory.
Header: “s/p MVC^b^, s/p chest removal” “status post MVC and chest tube removal, "	Error	No error	No error	Typographic omission of “tube” was mistaken for a clinical conflict.
“…image degradation in the low pelvis because of patient’s size, but no masses or fluid collections are seen.” “osteolytic and mixed osteosclerotic metastases are seen in the pelvic bones, most prominent at the right iliac…”	Error	Error	No error	Separate reporting of the pelvic cavity and pelvic bone was overlooked, and the statements were therefore flagged as contradictory.
Chest section: “The heart, pericardium, and great vessels are normal.” Abdomen section: “The IVC^c^ is markedly compressed; however, remains patent.”	Error	Error	No error	Separate reporting of chest and abdomen was overlooked, and the statements were therefore flagged as contradictory.
“The liver demonstrates normal morphology without signal dropout...There are numerous ill-defined lesions within the liver which are hypointense to the liver parenchyma...”	Error	Error	No error	Failure to distinguish overall morphology from focal lesions produced a false positive.

a All reports contained no actual errors. “Error” indicates false positive by framework; “No error” indicates correct assessment by framework.

b MVC: motor vehicle collision.

c IVC: inferior vena cava.

### Precision of LLM Frameworks

**Figure 4.**
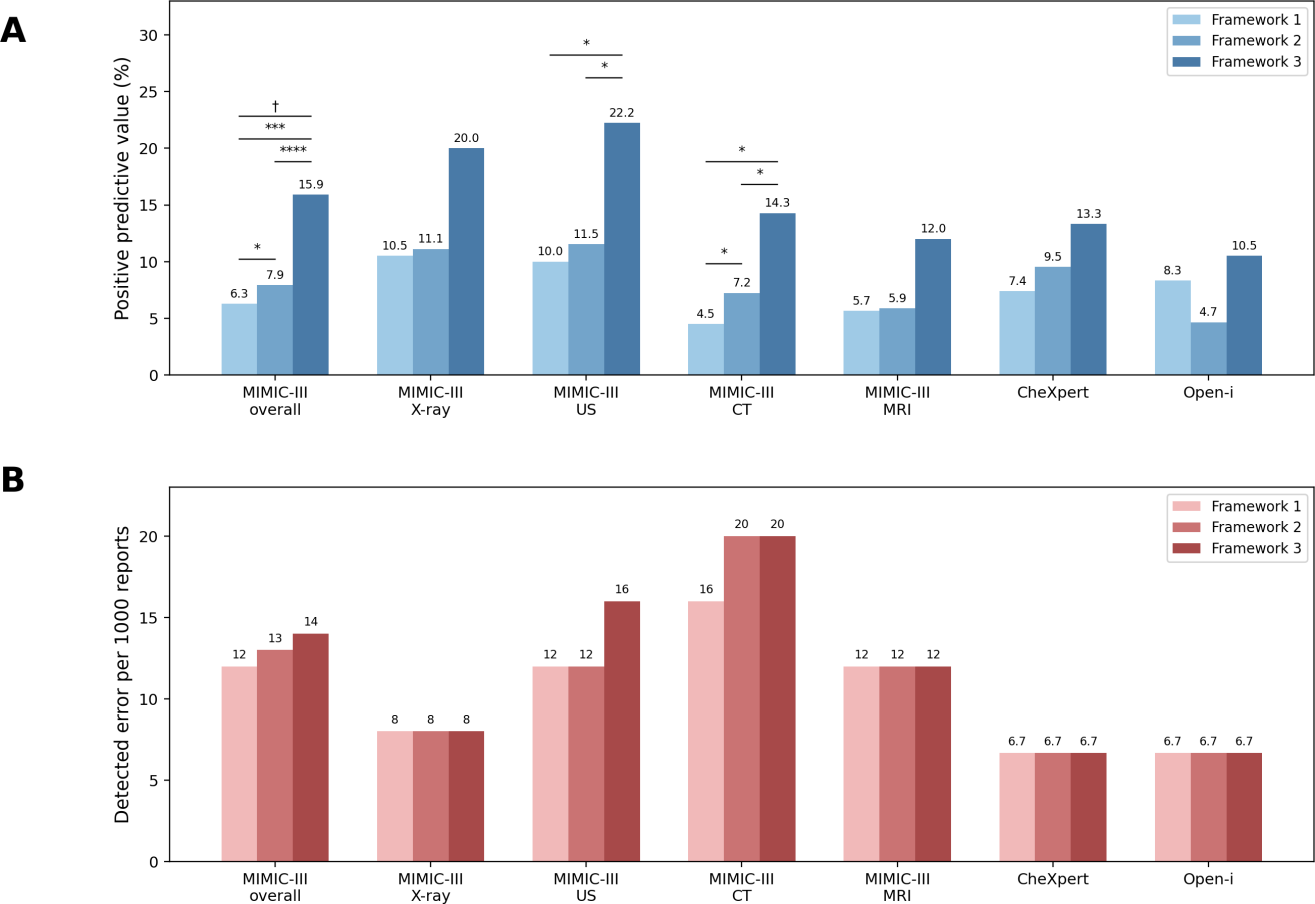
Performance comparison of the 3 error detection frameworks across the Medical Information Mart for Intensive Care III (MIMIC-III), CheXpert, and Open-i datasets. (A) Positive predictive value. (B) Detected errors per 1000 reports. Statistical significance was determined by the paired bootstrap test: *P*<.05, *P<.01, ***P<.001, ****P<.0001. Trend analysis was performed using the Cochran-Armitage test: †*P*<.05. CT: computed tomography; MRI: magnetic resonance imaging; US: ultrasound.

The precision of the LLM frameworks improved as the pipeline complexity increased (Table 3, Figure 4A). In framework 3, the overall PPV was 0.159 (95% CI 0.090‐0.252), compared with 0.079 in framework 2 and 0.063 in framework 1. The superiority of framework 3 over both framework 1 and framework 2 remained significant after multiple comparison correction (all paired-cluster bootstrap *P*<.001; all Holm-adjusted *P*<.001). A prespecified Cochran-Armitage trend test confirmed a significant upward trend in PPV across the 3 ordered frameworks (*P*=.02), indicating that successive refinements effectively reduced FP alerts.

**Table 3.** Positive predictive value (PPV) among 3 error detection frameworks across MIMIC-III^a^, CheXpert, and Open-i datasets.

Dataset, modality, and framework	TP^b^	FP^c^	PPV (95% CI)	*P* value^d^	Holm-adjusted *P* value^e^	Cochran-Armitage trend test *P* value
MIMIC-III						
Overall						
1	12	179	0.063 (0.033‐0.107)	.01	.01	.10
2	13	151	0.079 (0.043‐0.132)	<.001	<.001	—^f^
3	14	74	0.159 (0.090‐0.252)	<.001	<.001	—
X-ray						
1	2	17	0.105 (0.013‐0.331)	>.99	—	.52
2	2	16	0.111 (0.014‐0.347)	.29	—	—
3	2	8	0.200 (0.025‐0.556)	.29	—	—
Ultrasound						
1	3	27	0.100 (0.021‐0.265)	.42	—	.27
2	3	23	0.115 (0.024‐0.302)	.04	—	—
3	4	14	0.222 (0.064‐0.476)	.03	—	—
CT^g^						
1	4	85	0.045 (0.012‐0.111)	.02	—	.09
2	5	64	0.072 (0.024‐0.161)	.01	—	—
3	5	30	0.143 (0.048‐0.303)	.01	—	—
MRI^h^						
1	3	50	0.057 (0.012‐0.157)	.89	—	.39
2	3	48	0.059 (0.012‐0.162)	.09	—	—
3	3	22	0.120 (0.025‐0.312)	.10	—	—
CheXpert						
1	2	25	0.074 (0.009‐0.243)	.46	.79	.55
2	2	19	0.095 (0.012‐0.304)	.39	.79	—
3	2	13	0.133 (0.017‐0.405)	.26	.79	—
Open-i						
1	2	22	0.083 (0.010‐0.270)	.27	.80	.84
2	2	41	0.047 (0.006‐0.158)	.25	.80	—
3	2	17	0.105 (0.013‐0.331)	.57	.80	—

a MIMIC-III: Medical Information Mart for Intensive Care III.

b TP: true positive.

c FP: false positive.

d Two-sided paired-cluster bootstrap (10,000 replicates) *P* value—this row compares current framework with the next (row 1: framework 1 vs framework 2; row 2: framework 2 vs framework 3; row 3: framework 1 vs framework 3).

e Same comparisons as above.

f Not applicable.

g CT: computed tomography.

h MRI: magnetic resonance imaging.

The observed increase in precision was not accompanied by a reduction in TP detections (Table 4, Figure 4B). The overall DE/1k was 14 (95% CI 8‐23) for framework 3, compared with 13 (95% CI 7‐22) for framework 2 and 12 (95% CI 6‐21) for framework 1. None of the pairwise comparisons reached statistical significance (all *P*≥.84), indicating that framework 3 reduced FP flags without compromising error detection.

**Table 4.** Detected errors per 1000 radiology reports among 3 error detection frameworks across MIMIC-III^a^, CheXpert, and Open-i datasets.

Dataset, modality, and framework	Detected errors per 1000 (95% CI)	*P* value^b^	Holm-adjusted *P* value^c^	Cochran Q test *P* value
MIMIC-III				
Overall				
1	12 (6‐21)	>.99	>.99	.93
2	13 (7‐22)	>.99	>.99	—^d^
3	14 (8‐23)	.85	>.99	—
X-ray				
1	8 (1-29)	>.99	—	>.99
2	8 (1-29)	>.99	—	—
3	8 (1-29)	>.99	—	—
Ultrasound				
1	12 (2‐35)	>.99	—	.91
2	12 (2‐35)	>.99	—	—
3	16 (4‐40)	>.99	—	—
CT^e^				
1	16 (4‐40)	>.99	—	.93
2	20 (7‐46)	>.99	—	—
3	20 (7‐46)	>.99	—	—
MRI^f^				
1	12 (2‐35)	>.99	—	>.99
2	12 (2‐35)	>.99	—	—
3	12 (2‐35)	>.99	—	—
CheXpert				
1	7 (1-24)	>.99	>.99	>.99
2	7 (1-24)	>.99	>.99	—
3	7 (1-24)	>.99	>.99	—
Open-i				
1	7 (1-24)	>.99	>.99	>.99
2	7 (1-24)	>.99	>.99	—
3	7 (1-24)	>.99	>.99	—

a MIMIC-III: Medical Information Mart for Intensive Care III.

b McNemar test *P* value—this row compares current framework with the next (row 1: framework 1 vs framework 2; row 2: framework 2 vs framework 3; row 3: framework 1 vs framework 3).

c Same comparisons as above.

d Not applicable.

e CT: computed tomography.

f MRI: magnetic resonance imaging.

In the CheXpert and Open-i datasets, framework 3 achieved the highest PPVs (0.133 and 0.105, respectively; paired-cluster bootstrap *P*≥.26; Holm-adjusted *P*≥.79) and maintained identical DE/1k across frameworks (7 for both datasets; all *P*>.99), demonstrating robustness across diverse datasets. However, in the Open-i dataset, framework 2 yielded a lower PPV than framework 1—the only instance in which PPV did not increase monotonically with pipeline complexity. This exception may be due to the Open-i dataset already being extensively preprocessed, reducing the relative benefit of the first-pass LLM.

When framework 3 was executed using the o4-mini model instead of o3, the overall PPV significantly declined to 0.081 (*P*<.001; Table S6 in Multimedia Appendix 1), while the DE/1k decreased slightly to 12 without reaching statistical significance (*P*=.69; Table S7 in Multimedia Appendix 1).

### Analysis of FP Patterns

The distribution of FPs shifted distinctly toward semantic categories as the pipeline evolved (Table 5, Figure S2 in Multimedia Appendix 1). Superficial errors were effectively filtered by preprocessing, with header/metadata artifact cases decreasing from 3 out of 30 (10%) in framework 1 to 0% (0/30) in framework 2. However, rare preprocessing-induced artifact cases (1/30, 3%) emerged as a minor trade-off. Subsequently, structural mismatches were suppressed by the verifier step, resulting in the elimination of section/scope mismatch and lexical/abbreviation/typographical mismatch (0/30, 0%) in framework 3. Consequently, residual FPs in the final framework were predominantly concentrated in complex semantic categories, specifically clinically equivalent rephrasing (16/30, 53%) and unsupported discrepancy assertions (13/30, 43%).

**Table 5.** Characterization of false-positive categories across frameworks.

False-positive category	Definition	Framework 1 (n=30), n (%)	Framework 2 (n=30), n (%)	Framework 3 (n=30), n (%)
Header/metadata artifact	Header/history/technique/comparison text is treated as a body-text discrepancy.	3 (10)	0 (0)	0 (0)
Section/scope mismatch	Statements from different sections or anatomical scopes (eg, chest vs abdomen) are compared as if the same scope.	2 (7)	1 (3)	0 (0)
Lexical/abbreviation/typographical mismatch	Minor lexical differences (abbreviations, spelling, and formatting) are flagged as discrepancies.	2 (7)	1 (3)	0 (0)
Clinically equivalent rephrasing	Clinically acceptable wording is rewritten to a “preferred” term and the original is flagged as discrepant.	12 (40)	14 (47)	16 (53)
Unsupported discrepancy assertions	A discrepancy is asserted despite insufficient support (eg, wrong matching or compatible statements treated as conflict).	11 (37)	13 (43)	13 (43)
Preprocessing-induced artifact	Preprocessing (segmentation/normalization/removal) introduces artificial discrepancies.	0 (0)	1 (3)	1 (3)

### Operational Cost-Efficiency of LLM Frameworks

**Figure 5.**
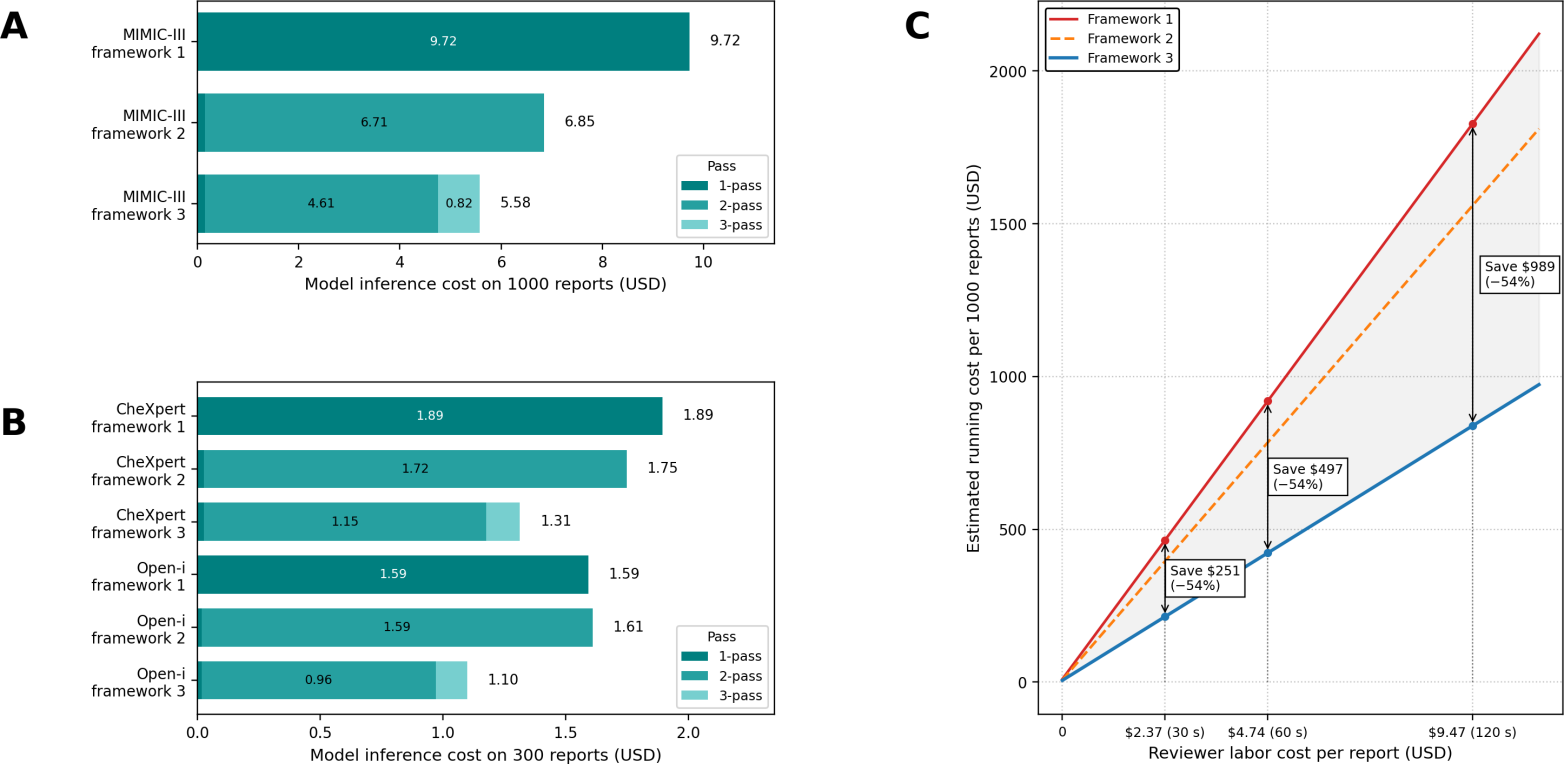
Cost analysis of the radiology report error detection frameworks and their component passes. (A) Model-only inference cost per 1000 reports in the Medical Information Mart for Intensive Care III (MIMIC-III) dataset. (B) Corresponding inference cost for 300 reports in the CheXpert and Open-i datasets. (C) Estimated total running cost per 1000 MIMIC-III reports plotted against reviewer labor cost per report. The analysis considers labor costs of US $2.37, US $4.74, and US $9.47, corresponding to review times of 30, 60, and 120 seconds, respectively. Annotations indicate the projected savings of framework 3 compared to framework 1.

The token counts for each pass are summarized in Table S8 in Multimedia Appendix 1. Framework 3 achieved the lowest model inference cost, at US $5.57 per 1000 reports, compared with US $9.72 and US $6.85 for frameworks 1 and 2, respectively—corresponding to cost reductions of approximately 42.6% and 18.5% relative to frameworks 1 and 2, respectively (Figure 5A). Framework 2 achieved most of its savings through token reduction via preprocessing, relative to framework 1. In framework 3, additional savings beyond those of framework 2 were primarily attributed to the FP verifier being triggered for only 88 candidate errors, rather than for all cases.

Based on the MGMA-derived rate (US $4.74/min), the estimated reviewer labor costs per report were US $2.37 (30 s), US $4.74 (60 s), and US $9.47 (120 s). Under these assumptions, framework 3 demonstrated a consistent advantage in reviewer labor cost. Compared to framework 1, framework 3 yielded estimated savings of US $251 (30-s scenario), US $497 (60-s scenario), and US $989 (120-s scenario) per 1000 reports in estimated running costs (Figure 5C).

A similar trend was observed in the CheXpert and Open-i datasets (Figure 5B), where framework 3 consistently demonstrated the lowest model inference costs per 300 reports (US $0.1374 and US $0.1271, respectively), compared with framework 1 (US $1.8943 and US $1.5930, respectively). However, in the Open-i dataset, framework 2 incurred a higher model inference cost than framework 1—representing the only instance in which model inference cost did not decrease with increased pipeline complexity.

## Discussion

The proposed 3-pass LLM framework improved precision and reduced estimated running costs without compromising error detection capability on datasets that approximate real-world error prevalence. On the MIMIC dataset, a PPV of 16% was achieved—more than twice that of a single-prompt, single-extraction baseline—while maintaining the detected error counts. The model inference cost decreased by 42.7% (US $9.72 vs US $5.57 per 1000 reports), and the number of alerts requiring human review declined by 54.2% (192 vs 88). These improvements remained robust across 2 independent datasets and within modality-specific subgroups.

Widely adopted clinical decision support systems, such as sepsis prediction or drug interaction alerts, typically exhibit low PPV in real-world settings because the cost of missing a critical event is unacceptable [26-28]. Radiology report error detection shares inherent challenges due to the low prevalence of errors in routine practice. To address these challenges, previous studies have often relied on synthetic error injection, based on the assumption that prevalence does not influence the sensitivity or specificity of the model [1,10,11]. However, this approach has notable limitations. Specifically, synthetic error injection may introduce bias in performance evaluation and error characterization, as the distribution and nature of injected errors may not accurately reflect real-world conditions. Furthermore, artificially inflating error prevalence can substantially overestimate the PPV, thereby misrepresenting the practical utility of the model in real-world scenarios. A low PPV—implying a high rate of false alarms—can increase the workload for radiologists and introduce potential biases for researchers conducting quality assurance on curated datasets; furthermore, it often induces distrust in the system, leading to “alert fatigue,” where alarms are habitually ignored [29]. Kim et al [3] demonstrated that few-shot prompting could improve GPT-4’s PPV to 0.12 on a dataset without injected errors. However, this improvement was derived from a post hoc analysis that reprompted only those cases previously identified as FPs, limiting the generalizability of the findings.

Thus, the proposed multipass architecture improves precision in real-world settings. This improvement is driven by 2 key components. First, a preprocessing LLM transforms raw radiology reports into cleaned, structured output before passing them to the primary LLM. During prompt tuning, we frequently observed that artifacts—such as embedded metadata, addenda, and page breaks—were misinterpreted as report content, thereby inflating FP rates. The preprocessor mitigates this issue by removing such noise, which not only reduces the likelihood of FPs but also decreases the input size for downstream tasks. However, these preprocessing prompts yielded minimal benefits on the already cleaned Open-i dataset. The FP analysis even identified a small number of cases where the preprocessing itself generated artifacts. Consequently, to achieve optimal performance, preprocessing strategies must be carefully adapted to the reporting conventions and dataset characteristics unique to each institution.

Second, the framework uses a detector-verifier cascade. When the detector is prone to FPs, separating detection and verification into 2 distinct steps allows the LLMs to complement each other: the detector prioritizes sensitivity, whereas the verifier enhances specificity. This arrangement parallels the tiered double-reading workflow commonly used in radiology; however, in this framework, the 2 LLMs perform the initial “double read,” and a human radiologist provides the final adjudication—effectively constituting a tiered triple read [30]. Prior evidence supports the benefits of task separation: in one study, 2 GPT-4 prompts for radiology report error detection were compared, revealing a trade-off between sensitivity and specificity, while the overall *F*_1_-score remained constant [3]. This suggests that, for error detection—where high sensitivity is essential—a 2-stage cascade that first maximizes sensitivity and then applies a highly specific verifier offers a more effective balance between error detection and alert fatigue.

The remaining FPs in this framework are largely confined to cases requiring deeper clinical context, highlighting an inherent limitation of LLMs in adjudicating nuanced clinical equivalence. This may partly explain why X-ray/ultrasound achieved relatively higher PPV than CT/MRI, as CT/MRI reports are typically longer and contain more complex, multifinding narratives. Importantly, this FP profile motivates a human-in-the-loop quality assurance design: AI can triage potential errors to shift the workflow from an “unguided” to a “targeted search,” while a secondary verifier layer filters out many structural FPs. Consequently, clinicians can focus their expertise on the smaller set of clinically ambiguous alerts that require high-level judgment, suggesting a complementary division of labor between AI speed and human expertise.

Successful clinical translation requires workflow-tailored integration. This framework is envisioned as an asynchronous background service that analyzes draft reports after initial dictation and surfaces only clinically meaningful report internal inconsistencies before final sign-off. To optimize the radiologist’s workload, notification timing should be adapted to the clinical context—for example, near-real-time alerts for emergency or intensive care unit studies, notifications before discharge for routine inpatient studies, and batched alerts before the next scheduled visit for outpatient studies. Initial deployment should be focused on high-acuity settings or predefined high-risk cohorts to maximize clinical benefits while minimizing alert fatigue; accumulated adjudication outcomes can then be leveraged for institution-specific refinement. To reduce the cognitive burden associated with alert review, it is essential that the error rationale be displayed alongside the flagged discrepancy, as implemented in the present framework. The seamless integration of these elements into the Picture Archiving and Communication System reading environment is equally critical to ensure that adjudication occurs within the radiologist’s existing workflow.

This study has some limitations. First, the cost analysis focused on estimated running costs (inference and labor) to allow for a direct comparison across frameworks. A comprehensive total cost of ownership analysis was not performed, as such an evaluation would require site-specific microcosting of integration, governance, and maintenance expenses. Additionally, because direct measurements of the power consumption of the closed-source model were not feasible, we used a token-processing charge as a surrogate. This approach was chosen to comparatively evaluate the superiority between frameworks, and actual measurements were beyond the scope of this study. The cost model also assumes a fixed per-alert review time; in practice, framework 3’s residual alerts—predominantly semantically complex cases, such as clinically equivalent rephrasing—may require longer adjudication than the structural artifacts filtered by earlier stages, potentially moderating the estimated labor savings. Future studies should aim to validate both actual computational usage and per-alert adjudication time in real-world deployment scenarios. Second, although the PPV doubled, the framework still generates an excessive number of alerts for a busy clinical workflow. In this study, typographical errors, along with all error candidates that could not be confirmed using the corresponding images, were conservatively classified as FPs; therefore, the reported PPV likely represents a lower bound. Even with this conservative estimate, the current precision remains insufficient for fully autonomous AI adoption. Many FPs resulted from the framework interpreting individual words too strictly, indicating a limitation in its ability to interpret clinical context effectively. Third, although the framework was validated on multiple datasets, real-world radiology reports vary substantially across institutions regarding templates, headers, and dictation styles, which may affect preprocessing reliability and shift downstream precision. Fourth, while the pipeline is architecturally modular, reported performance was obtained using specific proprietary models and may not directly translate to other architectures, such as open-source LLMs. Fifth, although the intended role is human-in-the-loop decision support rather than an autonomous agent, deployment entails ethical and legal considerations—including liability for missed errors and the risk of automation bias—necessitating strict oversight.

Future studies would greatly benefit from evaluating additional backbones, including locally fine-tuned LLMs and multimodal models that incorporate image context to broaden detectable error types. Additionally, quantifying end-to-end computational costs and incorporating institution-aware adaptation to mitigate heterogeneity in reporting styles in real deployment settings would be beneficial. Prospective evaluation in high-stakes, error-prone settings is warranted to validate practical utility and safety under human oversight.

In conclusion, the multipass LLM improved the precision and efficiency of radiology-report error detection in real-world, low-error prevalence settings. The framework demonstrates the feasibility of synergistic AI-radiologist collaboration and provides a cost-effective and scalable approach to AI-assisted quality assurance in both radiological practice and research.

## Supplementary material

10.2196/87368 Multimedia Appendix 1 Supplementary methodology and materials, including detailed large language model prompts, extended cost-efficiency derivations, and supplementary performance tables and figures.

## Abbreviations

AI: artificial intelligence
API: application programming interface
CT: computed tomography
DE/1k: detected errors per 1000 reports
FP: false positive
LLM: large language model
MGMA: Medical Group Management Association
MIMIC-III: Medical Information Mart for Intensive Care III
MRI: magnetic resonance imaging
PPV: positive predictive value
TP: true positive
