# Supplementary material for: Improving Radiology Report Error Detection Using a Multipass Large Language Model: Framework Development and Validation
Source: JMIR Med Inform. 2026 Jun 4;14:e87368. doi: 10.2196/87368 (PMC13235978; doi:10.2196/87368)
Supplement: Multimedia Appendix 1 [file medinform-v14-e87368-s001.docx]

**Multimedia Appendix**

**Improving Radiology Report Error Detection Using a Multi-Pass LLM: Framework Development and Validation**

**Supplementary Materials and Methods**

**Table S1. Descriptions of the error types used in this study**

| **Error types** |  |
| --- | --- |
| **Interpretive** |  |
| Substitution (1a) | Incorrect interpretation or misclassification in the Impression section about a specific finding mentioned in the Finding section. |
| Omission (1b) | Underreporting or missing the presence or severity of a specific finding in the Impression section compared with the Finding section. |
| Addition (1c) | Overreporting the presence or severity of a specific finding in the Impression section compared with the Finding section. |
| **Factual** |  |
| Discrepancy in location (2a) | Discrepancy in the location of the lesion within the same report |
| Discrepancy in numerical measurement (2b) | Discrepancy in numerical measurements within the same report |

**1. LLM prompt and parameter**

The prompts and parameters used for each evaluation pass are detailed below. All prompts were constructed using a task-context-output format. Model selection was based on OpenAI's official recommendations for optimal reasoning performance and cost-effectiveness at the time of study. All other parameters were set to default values.

Output formatting was enforced using JSON Schema mode[1], ensuring that every model response conformed to a predefined schema with required fields including findings/impression and error/error_reason. No JSON schema validation failures were observed during the evaluation process.

**1)1^st^ Pass LLM**

***Prompt:***

**Tasks**

1. Extract only content belonging to the *Findings* section (detailed observations) and the *Impression* / *Conclusion* / *Opinion* section (diagnostic interpretation).

2. If there is an *Addendum* or *Correction* section:

- Sentences that amend Findings ⇒ append to Findings.

- Sentences that amend the diagnostic interpretation ⇒ append to Impression.

- If ambiguous, append to Impression.

- Also, clearly mark it as "Addendum".

3. Discard every other section (history, technique, timestamps, signatures, headers, billing codes, etc.).

4. Replace every explicit calendar date with the literal token **[DATE]**.

5. Replace PHI with the literal token **[PHI]**.

(Output must follow the JSON schema exactly.)

{"Findings":"~", "Impression":"~"}

If either the Findings or Impression section is missing, set the corresponding value to "N/A".

***Parameter:***

*Json schema*:

{

"name": "preprocessing",

"strict": True,

"schema": {

"type": "object",

"properties": {

"findings": {"type": "string"},

"impression": {"type": "string"},

},

"required": ["findings", "impression"],

"additionalProperties": False,

},

}

Other parameters are set to defaults.

**2) 2^nd^ Pass LLM**

***Prompt:***

**Tasks**

Identify clinically significant errors based on the content within the provided radiology report.

1. Please read through the entire radiology report and understand the clinical scenario.

2. Identify any clinically significant errors in the report.

3. Limit errors to parts identifiable without images:

- Internal factual inconsistencies: Directly conflicting statements within the same radiology reports (e.g., conflicting laterality, measurements).

- Objective misinterpretations: Interpretations clearly and objectively contradicted by explicit statements in the Findings and Impression sections of the same radiology report.

(Output must follow the JSON schema exactly.)

If no error is found, return the JSON with

"error": "no error", "error_reason": "N/A".

If an error is found, return the JSON with

"error": "(cite erroneous statement from the report)", "error_reason": "(concise explanation; utilize quotes if necessary)".

***Parameter:***

*Json schema*:

{

"name": "error_report",

"strict": True,

"schema": {

"type": "object",

"properties": {

"error": {"type": "string"},

"error_reason": {"type": "string"},

},

"required": ["error", "error_reason"],

"additionalProperties": False,

},

}

Other parameters are set to defaults.

**3) 3^rd^ Pass LLM**

***Prompt:***

You will receive: 'radiology report JSON' and 'candidate error JSON'.

**Tasks**

Decide whether `candidate error JSON` is a TRUE clinically-significant internal error within `radiology report JSON` or a FALSE POSITIVE.

Guidelines to confirm an error:

- Objectivity: A true ERROR must be objectively incorrect, factually contradictory, or undeniably inaccurate.

- Clarity: The error must be so clear and obvious that ALL trained radiologists or medical professionals would unanimously agree it is incorrect.

- Clinical importance, differences in judgment, or disagreements about what should be included in the Impression or Findings DO NOT qualify as errors.

- Only contradictions or inaccuracies explicitly within the radiology report itself can qualify as errors. Differences between the report content and

provided clinical information, patient history, or external context must NEVER be considered errors.

(Output must follow the JSON schema exactly.)

If determined to be a FALSE POSITIVE, return JSON with:

"error": "no error", "error_reason": "N/A".

***Parameter:***

*Json schema*:

{

"name": "error_report",

"strict": True,

"schema": {

"type": "object",

"properties": {

"error": {"type": "string"},

"error_reason": {"type": "string"},

},

"required": ["error", "error_reason"],

"additionalProperties": False,

},

}

**2. Efficiency evaluation**

***1) Notation and units***

We let *N*_in,k_ ​ and *N*_out,k_ ​ denote the numbers of input and output tokens, respectively, for the k‑th model pass. The constants *P*_in_ and *P*_out_ ​ represent the vendor’s charge per input and output token. The set *E* collects every report that was flagged for human review—regardless of whether the flag was ultimately adjudicated as a true or a false error—and ∣*E*∣ therefore equals the total count of such flags. Finally, *C*_review_ denotes the mean fee paid to a radiologist for adjudicating a single flagged report. A complete list of notation is provided in Supplementary Table 1, and the model‑specific prices are summarised in Supplementary Table 2.

**Table S2.** Definitions of symbols and corresponding units used in the cost‑efficiency analysis.

| Symbol | Definition | Unit |
| --- | --- | --- |
| *N*_in,k_ | Input tokens processed in model pass *k* | tokens |
| *N*_out,k_ | Output tokens generated in model pass *k* | tokens |
| *P*_in_ | Price per input token (model‑specific) | USD / token |
| *P*_out_ | Price per output token (model‑specific) | USD / token |
| *E* | Set of all reports that the model flagged for human review |  |
| *C*_review_ | Average fee paid for reviewing one flagged report | USD / report |

**Table S3.** Supplementary Table 2. Processing prices of OpenAI large‑language models (as of 10 May 2025)

| Model | Input price (USD / 1 K tokens) | Output price (USD / 1 K tokens) |
| --- | --- | --- |
| gpt‑4.1‑nano | $0.10 | $0.40 |
| o3 | $10.00 | $40.00 |
| o4‑mini | $1.10 | $4.40 |

***2) Derivation of cost components***

The **model-inference cost** accumulates over all *K* passes:

**
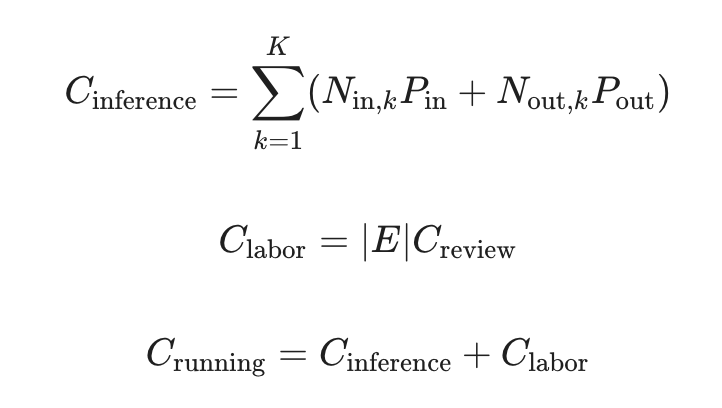
** –  Eq. S1

The **reviewer-labor cost** converts is calculated by multiplying the number of reports flagged for review by the average fee paid per reviewed report.

**
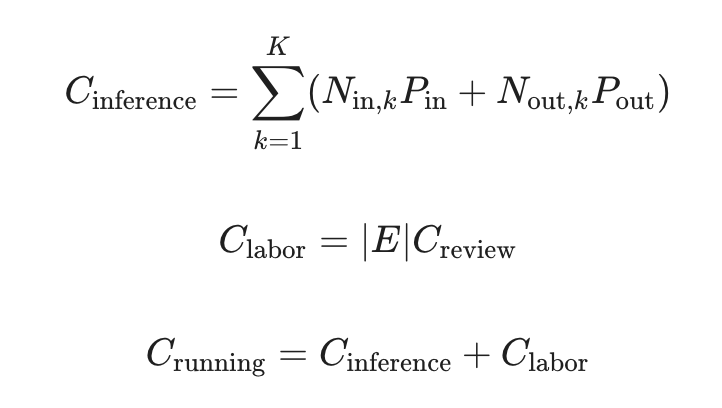
** – Eq. S2

The **estimated running cost** is the sum of two components:

**
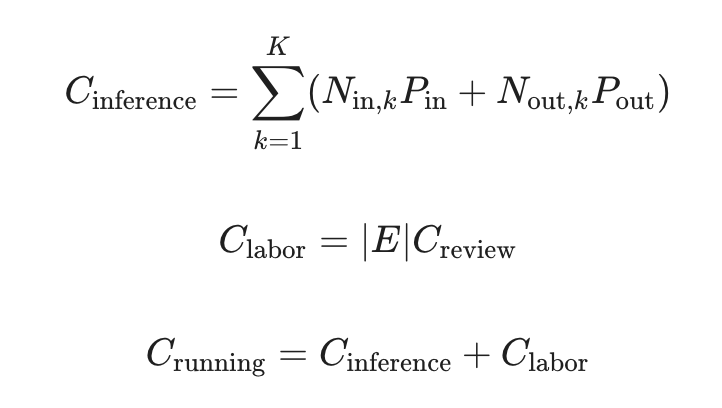
** – Eq. S3

**Supplementary Results**

**Table S4.** True-positive detection errors identified by Framework 3 in the MIMIC-III, CheXpert, and Open-i datasets

| **Report** | **Description** | **Types** |
| --- | --- | --- |
| **MIMIC-III** |  |  |
| FINDINGS: AP single view of the chest has been obtained with patient in  sitting semi-upright position.  ...   A right-sided PICC line is now identified, seen to terminate overlying the  right-sided mediastinal structures at the level 3 cm below the carina. This  is compatible with the lower third of the *IVC*.  ... | CHEST PORT. LINE PLACEMENT | 2a |
| ...  The focal ill-defined opacity in this region noted on the  prior film of [**2187-8-10**], has resolved. A jugular CV line is in  proximal SVC. No pneumothorax. *Bilateral pleural effusions.*    IMPRESSION: Resolution of peripheral ill-defined opacity in left upper lobe.  Persistent spiculated density in this location. *No pneumothorax or bilateral  pleural effusions.* ?? Scarring ?? right lung apex. | CHEST (PORTABLE AP) | 1b |
| There is an *echogenic vessel in the right thalamus*. This is seen on the  sagittal views. It is not changed from the prior examination and likely  represents a calcified lenticulostriate vessel.  ...    IMPRESSION: Calcified v*essel in the left thalamus* is unchanged. Examination  is normal otherwise. | NEONATAL HEAD PORTABLE | 2a |
| ...  On the *right, moderate plaque is seen at the upper portion of the common  carotid artery (CCA) as well as at the origins of the internal (ICA) and  external (ECA) carotid arteries. The peak systolic velocities (PSVs) are 160,  100 and 148 cm/sec, respectively.* The *right ICA to CCA PSV ratio is 1.6.* ...   CONCLUSION:  1. Bilateral moderate plaque in the ICAs, with associated luminal narrowing  of 60 to 69% (in diameter) on the right and 40 to 59% on the left. ... | CAROTID SERIES COMPLETE | 2b |
| ...  On the left, there is wall thickening of the common carotid artery, with some  echogenic deposits with posterior acoustic shadowing (calcifications). The  stent is visualized. T*he peak systolic velocities in the internal, external  common carotid arteries are 84, 83 and 269 cm/second, respectively. The left  internal to common carotid artery velocity ratio is 1.01.*   ...    CONCLUSION: No significant change as compared to the two previous  examinations. | CAROTID SERIES COMPLETE | 2b |
| ...  On the right systolic/end diastolic velocities of the ICA proximal, mid and  distal respectively are 87/15, 88/22, 72/17, cm/sec. CCA peak systolic  velocity is 110/14 cm/sec. ECA peak systolic velocity is 89 cm/sec. The  ICA/CCA ratio is .80. *These findings are consistent with no stenosis.*  ...    Impression: *Right ICA stenosis.*  Left ICA stenosis 40-59%. | CAROTID SERIES COMPLETE | 1c |
| ...  No newly developed mass effect with  persistent *approximately 9 mm leftward midline deviation from known extensive right temporal mass.* No other areas of high attenuation that would be  concerning for hemorrhage. There is also noted to be sulcal effacement by  this mass with associated and compression of the right lateral ventricle. ...    IMPRESSION: Several areas of high attenuation seen within the right temporal partial lobectomy bed done could be related to mild post op hemorrhage . . No new mass effect with *stable rightward midline shift by approximately* stable leftward midline shift by approximately 9 mm.  ... | CT HEAD W/O CONTRAST | 2a |
| ...  There is periventricular hypoattenuation consistent with small vessel ischemic disease. The right mastoid, middle ear and *right sphenoid are opacified consistent with otitis media as well as mastoiditis.* Also noted is mild  opacification of the left mastoid air cells as well as the right sphenoid.  This is likely secondary to endotracheal intubation.  ...   IMPRESSION:  ...  2. Opacification of the right mastoid, *both sphenoid sinuses* and right middle  ear cavity likely representing otitis media and mastoiditis secondary to ETT  placement. | CT HEAD W/O CONTRAST | 2b |
| ...  Please note that this exam was not tailored for subdiaphragmatic evaluation.  Limited evaluation of the included upper abdomen displayed some radiopaque substances within the gastric lumen likely related to pill ingestion and a *large conglomerate 26 x 51-mm calcified mass within the peritoneum* displacing the adjacent diaphragmatic crus which is most consistent with calcified nodes from treated lymphoma.   ...   IMPRESSION: ...  2. Mild post-radiation changes involving the paramediastinal upper lobes  consistent with known treated lymphoma. Large conglomerate calcified  *retroperitoneal mass* also resultant of treated lymphoma. ... | CT CHEST W/O CONTRAST | 2a |
| ...  There is a new 1.2 x 0.9 cm *nodule in the superior segment of the left lower lobe* (2:21).There is also a new area of ground-glass opacity in the medial aspect of the right middle lobe measuring approximately 1.0 x 0.3 cm (3:28). Previously identified pulmonary nodules in bilateral lungs are otherwise stable (3:24, ,30, 34, 35, 46, 50) .  ...    IMPRESSION:  1. New 1.2 x 0.9 cm nodule in the *superior segment of the left upper lobe* and  new area of ground-glass opacity in the medial aspect of the right middle  lobe, as well as multiple stable pulmonary nodules likely metastatic in  nature. ... | CT CHEST W/O CONTRAST | 2a |
| ...  Interval evolution of bilateral cerebellar hemispheric infarcts.  Unchanged small right frontal intraparenchymal hemorrhage surrounding a right  frontal approach ventriculostomy catheter which ends at the foramen of [**Last Name (un) 7030**]. The known diffuse bilateral subarachnoid hemorrhages as well as the intraventricular hemorrhage layering in the occipital horns have slightly decreased in density. *New acute intracranial hemorrhage and no acute cerebral infarction.* Unchanged mucosal thickening of the left maxillary sinus, sphenoid sinus, ethmoid and frontal sinuses. The mastoid air cells are clear.    IMPRESSION: ...  5. *No new acute intracranial hemorrhage or new acute infarction.* | CT HEAD W/O CONTRAST | 1b |
| MR brain with contrast: Within the [**Doctor Last Name 37**] matter of the *left parietal lobe is a small thick rim enhancing 7 mm round lesion* with moderate amount of associated vasogenic edema. Signal is slightly hyperdense to [**Doctor Last Name 37**] matter on T2 imaging with a hypodense surrounding rim.  ...  IMPRESSION:  1. Thick rim enhancing *7 mm right parietal lesion* with associated edema that  represents abscess versus neoplasm. Correlation with outside CT to determine  presence of calcification is advised, and if access to outside CT is not  available, reimaging is advised for further characterization. ... | MR HEAD W & W/O CONTRAST | 2a |
| ...  The right Posterior Inferior Cerebellar artery is visualized on the prior  CTA; left Anterior inferior cerebellar artery is not seen. The P1 segments of  the *posterior cerebellar arteries* are hypolastic with prominent posterior  communicating arteries on both sides representing fetal pattern.    IMPRESSION:  1. Multiple small acute infarcts within the left MCA territory with associated  stenosis of the M2 branches of the left middle cerebral artery, better  evaluated on prior CTA.  2. Mild thickening of the ligaments posterior to the dens without cord  compression- can be degenerative or inflammatory- to correlate with past  history. | MR HEAD W/O CONTRAST | 2a |
| ...  There is mildly increased signal, in the cortex in the left frontal lobe,  (series 6, image 20), superiorly near the vertex, with increased signal on the  diffusion-weighted sequence. In addition, *there are two punctate foci, noted  adjacent to the cortex, in the left frontal and in the right parietal lobes*  (series 302, image 22). These do not have definitively convincing decreased  signal on the ADC sequence.  ...    IMPRESSION:  1. Small areas of increased signal on the FLAIR and the DWI in the left  frontal lobe, the *punctate focus in the left parietal lobe*, which may relate  to acute-subacute infarction or edema. Given their small size, accurate  assessment is somewhat limited, as these are not clearly identifiable on the  ADC sequence. To correlate clinically and if necessary, followup can be  considered. ... | MR HEAD W/O CONTRAST | 2a |
| **CheXpert** |  |  |
| ...  Right internal jugular sheath with central line, endotracheal tube, nasogastric  tube, and feeding tube are unchanged. There is no evidence of  pneumothorax. *Compared with the prior examination, there has been  slight increase in interstitial pulmonary edema.* However, there is  decreased aeration at the left base. ...   IMPRESSION: ...  3. *DECREASED INTERSTITIAL PULMONARY EDEMA.* |  | 1b |
| ... The lung parenchyma is clear. There *are no pleural or significant bony   abnormalities.*    IMPRESSION: ...  *Bilateral moderate pleural effusions.*  No focal infiltrate. |  | 1c |
| **Open-i** |  |  |
| ... There has been interval sternotomy with intact midline sternotomy XXXX.  *The heart is near top normal in siz*e with unfolding of the aorta.  ...   IMPRESSION:  *Cardiomegaly*, however no acute cardiopulmonary findings. |  | 1c |
| ...  The cavity and the left upper lobe has decreased in size.   Bilateral apical bullae and parenchymal scars are unchanged.  ...    IMPRESSION:  Bullous disease and upper lobe scars. Decreasing right upper lobe cavity. |  | 2a |

**Table S5.** Per-framework true-positive error type distribution in the MIMIC-III dataset

| **Error type** | **Framework 1** | **Framework 2** | **Framework 3** | **Total** |
| --- | --- | --- | --- | --- |
| *Interpretive errors* |  |  |  |  |
| Substitution (1a) | 0 | 0 | 0 | 0 |
| Omission (1b) | 1 | 2 | 2 | 2 |
| Addition (1c) | 1 | 1 | 1 | 1 |
| *Factual errors* |  |  |  |  |
| Location discrepancy (2a) | 8 | 8 | 9 | 9 |
| Numeric discrepancy (2b) | 2 | 2 | 2 | 2 |
| **Total** | **12** | **13** | **14** | **14** |

Values represent number of true-positive errors detected. Errors detected by each framework constitute a strict subset of those detected by the next framework.

**Table S6.** Positive predictive values between o3 and o4-mini models in the MIMIC-III dataset

| **Dataset** | **Modality** | **Framework 3 o3 PPV (95% CI)** | **Framework 3 o4-mini PPV (95% CI)** | **p-value⁺** |
| --- | --- | --- | --- | --- |
| MIMIC-III |  |  |  |  |
|  | Overall | 0.159 (0.097-0.250) | 0.081 (0.047-0.136) | <.001 |
|  | X-ray | 0.200 (0.057-0.510) | 0.091 (0.025-0.278) | 0.278 |
|  | Ultrasound | 0.222 (0.090-0.452) | 0.160 (0.064-0.347) | 0.097 |
|  | CT | 0.143 (0.063-0.294) | 0.075 (0.030-0.179) | 0.015 |
|  | MR | 0.120 (0.042-0.300) | 0.042 (0.012-0.140) | 0.094 |

⁺ Two-sided paired cluster bootstrap (1000 replicates) p-value. Abbreviations: TP, true positives; FP, false positives; PPV, positive predictive value; CI, confidence interval;

**Table S7.** Absolute true-positive rates between o3 and o4-mini models in the MIMIC-III dataset

| **Dataset** | **Modality** | **LLM3_o3 aTPR (95% CI)** | **LLM3_o4-mini aTPR (95% CI)** | **p-value⁺** |
| --- | --- | --- | --- | --- |
| MIMIC-III |  |  |  |  |
|  | Overall | 0.014 (0.008-0.023) | 0.012 (0.007-0.021) | 0.693 |
|  | X-ray | 0.008 (0.002-0.029) | 0.008 (0.002-0.029) | 1.000 |
|  | Ultrasound | 0.016 (0.006-0.040) | 0.016 (0.006-0.040) | 1.000 |
|  | CT | 0.020 (0.009-0.046) | 0.016 (0.006-0.040) | 0.737 |
|  | MR | 0.012 (0.004-0.035) | 0.008 (0.002-0.029) | 0.653 |

⁺ McNemar test p-value. Abbreviations: aTPR, absolute true-positive rate; CI, confidence interval;

**Table S8.** Inference cost per pass among three error detection frameworks across MIMIC-III, CheXpert, and Open-i datasets

| **Dataset** | **Framework** | **# Passes** | **Model** | **N calls** | **Input tokens (×10³)** | **Output tokens (×10³)** | **Input/call** | **Output/call** | **Cost (USD)** |
| --- | --- | --- | --- | --- | --- | --- | --- | --- | --- |
| MIMIC-III |  |  |  |  |  |  |  |  |  |
|  | 1 | 1 | o3 | 1000 | 868.532 | 25.925 | 868.53 | 25.92 | 9.7223 |
|  | 2 | 1 | 4.1-nano | 1000 | 681.532 | 187.901 | 681.53 | 187.9 | 0.1433 |
|  | 2 | 2 | o3 | 1000 | 575.901 | 23.72 | 575.9 | 23.72 | 6.7078 |
|  | 3 | 1 | 4.1-nano | 1000 | 681.532 | 187.071 | 681.53 | 187.07 | 0.143 |
|  | 3 | 2 | o3 | 1000 | 369.071 | 23.089 | 369.07 | 23.09 | 4.6143 |
|  | 3 | 3 | o3 | 88 | 50.175 | 8.023 | 570.17 | 91.17 | 0.8227 |
| CheXpert |  |  |  |  |  |  |  |  |  |
|  | 1 | 1 | o3 | 300 | 166.082 | 5.837 | 553.61 | 19.46 | 1.8943 |
|  | 2 | 1 | 4.1-nano | 300 | 109.982 | 35.635 | 366.61 | 118.78 | 0.0253 |
|  | 2 | 2 | o3 | 300 | 152.035 | 5.087 | 506.78 | 16.96 | 1.7238 |
|  | 3 | 1 | 4.1-nano | 300 | 109.982 | 36.58 | 366.61 | 121.93 | 0.0256 |
|  | 3 | 2 | o3 | 300 | 91.18 | 5.968 | 303.93 | 19.89 | 1.1505 |
|  | 3 | 3 | o3 | 15 | 7.579 | 1.539 | 505.27 | 102.6 | 0.1374 |
| Open-i |  |  |  |  |  |  |  |  |  |
|  | 1 | 1 | o3 | 300 | 139.682 | 4.904 | 465.61 | 16.35 | 1.593 |
|  | 2 | 1 | 4.1-nano | 300 | 83.582 | 20.498 | 278.61 | 68.33 | 0.0166 |
|  | 2 | 2 | o3 | 300 | 136.898 | 5.615 | 456.33 | 18.72 | 1.5936 |
|  | 3 | 1 | 4.1-nano | 300 | 83.582 | 20.546 | 278.61 | 68.49 | 0.0166 |
|  | 3 | 2 | o3 | 300 | 75.146 | 5.091 | 250.49 | 16.97 | 0.9551 |
|  | 3 | 3 | o3 | 19 | 6.719 | 1.497 | 353.63 | 78.79 | 0.1271 |

**Supplementary References**

1. OpenAI Platform [Internet]. [cited 2025 Jun 11]. Available from: https://platform.openai.com/docs/guides/structured-outputs/examples?api-mode=responses


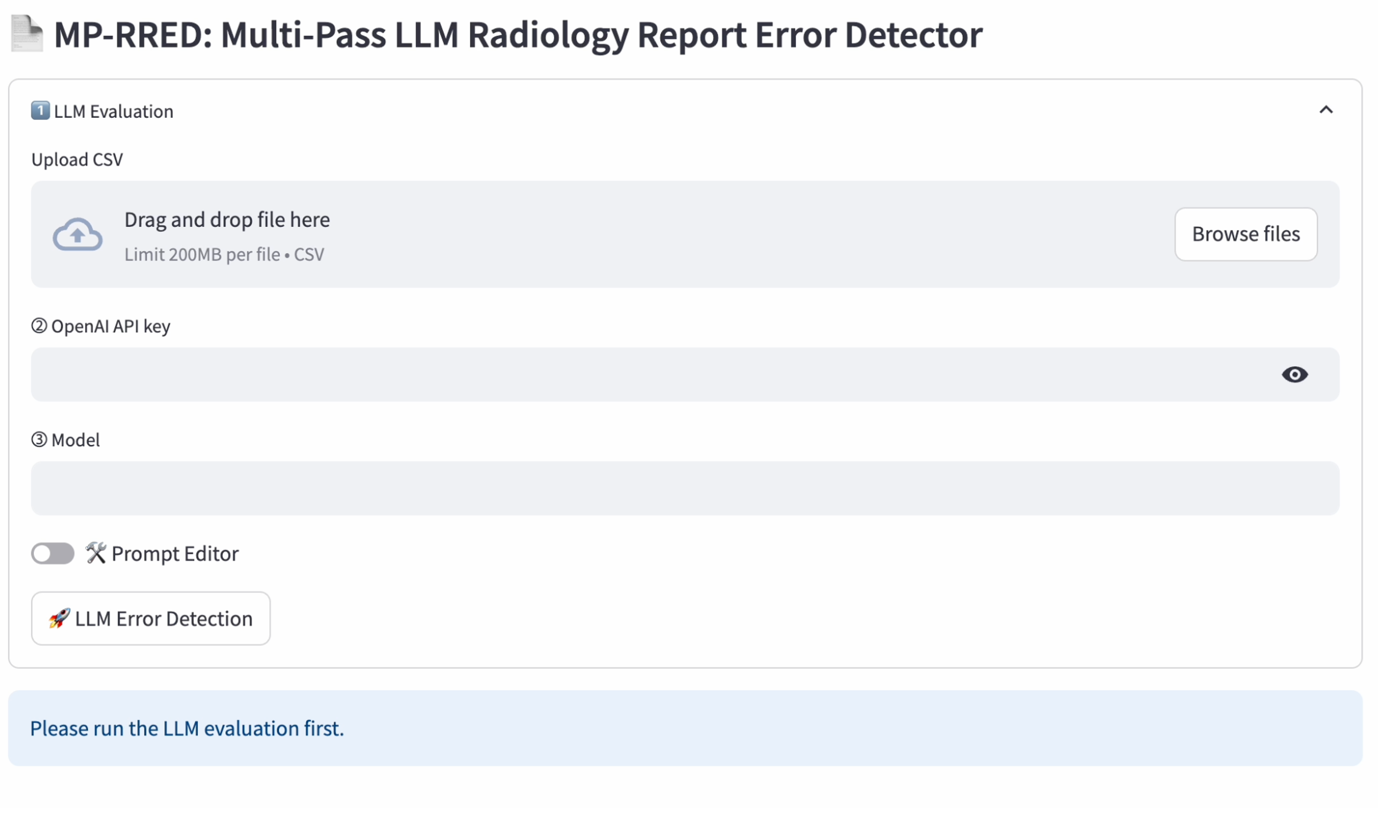


**Figure S1**. User interface for the multi-pass large language model radiology report error detector. The evaluation console allows users to upload a CSV file of radiology reports and initiate error analysis.


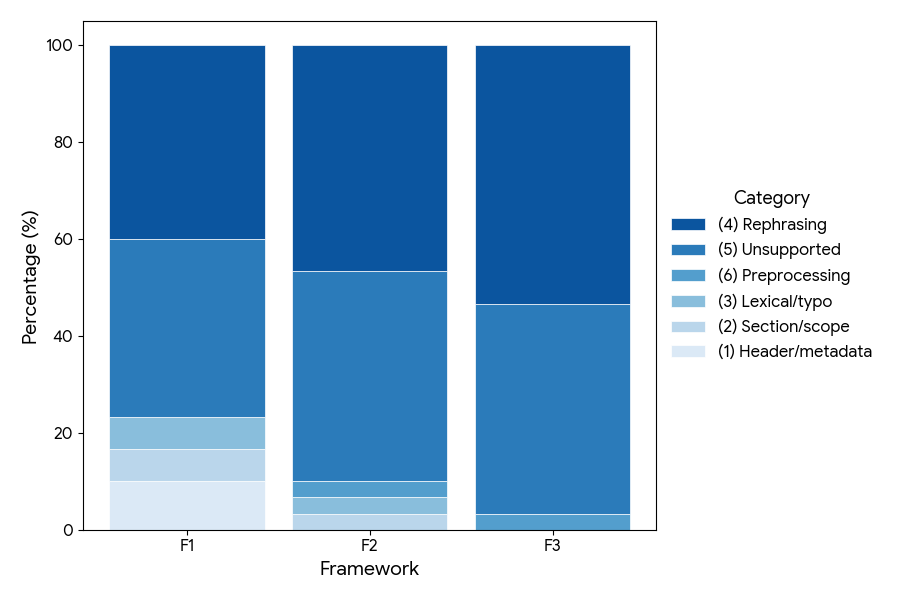


**Figure S2.** False-positive pattern variations across frameworks
